# Disparities in Telemedicine Success and Their Association With Adverse Outcomes in Patients With Thoracic Cancer During the COVID-19 Pandemic

**DOI:** 10.1001/jamanetworkopen.2022.20543

**Published:** 2022-07-07

**Authors:** Najeff Waseem, Mary Boulanger, Lisa R. Yanek, Josephine L. Feliciano

**Affiliations:** 1 Johns Hopkins Sidney Kimmel Cancer Center, Baltimore, Maryland; 2 Department of Internal Medicine, Johns Hopkins University Hospital, Baltimore, Maryland

## Abstract

**Importance:**

Disparities in access to telemedicine were identified at the onset of the COVID-19 pandemic, but the consequences of these disparities are not well characterized.

**Objective:**

To investigate factors associated with successfully accessing and completing telemedicine visits and the association between telemedicine visit success and clinical outcomes among patients with thoracic cancer.

**Design, Setting, and Participants:**

This retrospective cohort study included patients who attended outpatient visits at the thoracic oncology division of Johns Hopkins Medical Institute in Baltimore, Maryland, from March 1 to July 17, 2020.

**Main Outcomes and Measures:**

Associations of age, sex, race, ethnicity, insurance status, marital status, zip code, type of cancer, cancer stage, and type of therapy with telemedicine visit success (defined as completed visits with synchronous audio-video connection) and of visit success status with changes in therapy and odds of emergency department and urgent care visits, hospitalizations, and death were assessed using χ^2^ and Fisher exact tests and are reported as odds ratios (ORs).

**Results:**

A total of 720 patients and 1940 visits with complete data were included in the analysis; the median patient age was 65.7 years (range, 54.7-76.7 years), and 384 (53.33%) were male. Of the 1940 visits, 679 (35.00%) were in person and 1261 (65.00%) were telemedicine. Of the telemedicine visits, 717 (56.86%) were successful and 544 (43.14%) were unsuccessful. Patients who were Black (OR, 0.62; 95% CI, 0.41-0.95), had Medicaid (OR, 0.38; 95% CI, 0.18-0.81), or were from a zip code with an elevated risk of cancer mortality (OR, 0.51; 95% CI, 0.29-0.90) were less likely to have successful telemedicine visits than to have unsuccessful visits. Patients with at least 1 unsuccessful telemedicine visit had higher likelihood of an emergency department (OR, 2.73; 95% CI, 1.42-5.22) or urgent care (OR, 4.50; 95% CI, 2.41-8.41) visit or hospitalization (OR, 2.37; 95% CI, 1.17-4.80). Similarly, patients who had no successful telemedicine visits and for whom more than 1 telemedicine visit was scheduled had a higher likelihood of an emergency department (OR, 3.43; 95% CI 1.80-6.52) or urgent care (OR, 4.24; 95% CI 2.24-8.03) visit or hospitalization (OR, 4.19; 95% CI 2.17-8.10). Patients with all successful telemedicine visits (OR, 0.52; 95% CI, 0.30-0.90) or only 1, unsuccessful visit (OR, 0.32; 95% CI, 0.13-0.75) had lower odds of death compared with patients seen in-person only. Starting a new therapy was associated with lower odds of having a telemedicine visit vs an in-person visit (OR, 0.49; 95% CI, 0.37-0.64) and higher odds of a successful telemedicine visit vs an unsuccessful telemedicine visit (OR, 1.90; 95% CI, 1.28-2.82).

**Conclusions and Relevance:**

In this cohort study, patients with thoracic cancer who were Black, had Medicaid, or were from a zip code with a high risk of cancer mortality had increased odds of unsuccessful telemedicine visits compared with their counterparts and unsuccessful telemedicine visits were associated with worse clinical outcomes compared with successful visits. These findings suggest that more work is needed to improve telemedicine access for disadvantaged patients.

## Key Points

**Question:**

Are clinical and sociodemographic factors associated with disparities in successful completion of telemedicine visits, and are unsuccessful telemedicine visits associated with poorer clinical outcomes compared with successful visits?

**Findings:**

In this cohort study of 720 US patients with thoracic cancer during the COVID-19 pandemic, patients who were Black and/or had Medicaid had a significantly higher odds of unsuccessful telemedicine visits than their counterparts. Having at least 1 unsuccessful telemedicine visit was associated with higher odds of emergency department and urgent care visits and hospitalizations compared with having all successful telemedicine visits.

**Meaning:**

The findings suggest that there are disparities in telemedicine access among patients at risk of adverse health outcomes and that unsuccessful telemedicine visits are associated with poor long-term health outcomes.

## Teaser

This cohort study examines associations between patient sociodemographic factors, successful completion of telemedicine visits, and clinical outcomes among patients with thoracic cancer during the COVID-19 pandemic.

## Introduction

In 2020, the practice of medicine, including oncology, underwent 2 large paradigm shifts. The first, owing to the COVID-19 pandemic, was the rapid adoption and use of telemedicine to replace many in-person visits.^1^ The second, in response to the killing of George Floyd and the associated social justice protests, was a reexamination of the ways in which medical institutions may contribute to disparities in care.

The list of disparities in cancer care is exhaustive, and many have been associated with race and ethnicity, economic status, or insurance status. Disparities can occur at any point in a patient’s health care experience and include those in factors associated with development of cancer, screening, access to care and treatment, and clinical outcomes. A primary example of inferior outcomes in cancer care is a persistent lower rate of survival among Black patients compared with White patients across most cancer subtypes.^2^ The COVID-19 pandemic has also been associated with exacerbated health care disparities; Black patients with cancer were observed to be more likely to contract COVID-19 than were White patients and had worse outcomes, including higher rates of both hospitalization and death.^3^

To reduce health care access disparities, telemedicine efforts and digital health care visits have been increasingly implemented and studied in the past decade as a means to improve patient access to care.^4^ Most oncology patients surveyed reported high satisfaction with telemedicine services, and during the COVID-19 pandemic, synchronous audio-video visits were rapidly adopted as the standard alternative to in-person visits.^5^ Although there is limited evidence on the benefits of synchronous audio-video visits compared with audio-only visits, potential advantages include the ability to see patients, build rapport, and use nonverbal communication.^6,7^

The use of telemedicine and health care technology has also been shown to be a substantial source of disparity in health care access, and this has often been attributed to a larger problem known as the “digital divide.”^8^ Many studies have shown that Black, Latinx, non–English speaking, and older patients and those with Medicare or Medicaid are less likely to use telemedicine.^9,10,11^ Specifically, these same groups are also less likely to use a video visit^12,13^ and are overrepresented among US individuals who lack broadband internet access.^14^ Patients who have had audio-only visits are significantly less likely than those who have had audio-video visits to prefer using telemedicine for discussing their care plan and long-term health issues.^15^

We sought to identify disparities in telemedicine use and to explore whether access to and success of telemedicine efforts were associated with clinical outcomes in a large thoracic oncology practice. We conducted a retrospective cohort study of patients with thoracic cancer seen in-person or via video or telephone at the onset of the COVID-19 pandemic. We also assessed short-term outcomes during this period with regard to treatment decisions during each visit and rates of emergency department (ED) and urgent care visits, hospitalization, and death.

## Methods

**Figure 1.**
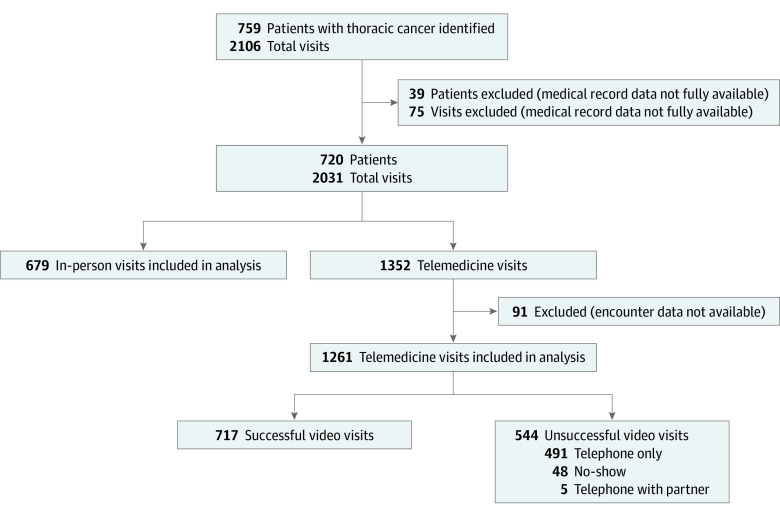
Patients and Visits Included in the Study

We identified patients with thoracic malignant tumors (Figure 1) treated at Johns Hopkins Medical Institute who had outpatient clinic visits between March 1 and July 17, 2020. Patients were selected after all medical oncology visits in the study period and were identified using Epic, the electronic medical record. Patients or visits were excluded if they did not have at least 1 completed office note with a medical oncologist. We categorized telemedicine visits as successful if a patient completed the entire scheduled visit with video capability or as unsuccessful if the visit was conducted via telephone without video or the patient had a no-show or missed encounter. There were not enough no-show encounters to conduct a separate analysis, and audio-only visits were categorized as unsuccessful because the institutional standard was to replace in-person visits with synchronous audio-video visits. We performed a retrospective review of medical records for demographic data; success of telemedicine visits; changes in therapy; deaths; and the number of hospitalizations, urgent care visits, and ED visits from March 1 to August 15, 2020. We also defined a subset of 9 zip codes (eFigure in the Supplement) in East Baltimore, Maryland, as high risk because they had previously been identified through our institutional cancer registry as having elevated rates of cancer mortality compared with other zip codes in Baltimore. The institutional review board at Johns Hopkins approved this study and waived informed consent because the study was retrospective. The study followed the Strengthening the Reporting of Observational Studies in Epidemiology (STROBE) reporting guideline.

Demographic factors examined included age, sex, race, ethnicity, insurance status, marital status, and zip code. Sex, race, and ethnicity were classified by patients in their patient portals, with race designations including Black, White, and other (American Indian, Asian, Native Hawaiian or Pacific Islander, or other unspecified) and ethnicity including Hispanic or Latino or not Hispanic or Latino. The clinical outcomes measured included ED visits, urgent care visits, hospitalizations, and death. We categorized patients into 6 groups with regard to types of visits: in-person visits only, all successful telemedicine visits, mixed success with only 1 unsuccessful telemedicine visit and the rest successful, mixed success with more than 1 unsuccessful telemedicine visit and the rest successful, no successful telemedicine visits among more than 1 telemedicine visit, and no successful telemedicine visits but only 1 telemedicine visit overall. The changes in therapy were obtained from physician office notes in the electronic medical record (eg, continuing the patient’s current therapy was categorized as maintenance, and disease progression requiring a new therapy or having an adverse effect requiring a change in treatment was categorized as a switch in therapy).

### Statistical Analysis

We evaluated the association of demographic factors and clinical data, such as type and stage of cancer and current treatment, with the success of telemedicine visits. We also assessed the association between success of telemedicine visits and clinical outcomes and changes in therapy after visits. Missing data were minimal; thus, a complete case analysis was performed. We used χ^2^ or Fisher exact tests, as appropriate, and present odds ratios (ORs) with 95% CIs. To account for nonindependence of patient visits, we used generalized estimating equation (GEE) multivariate logistic regression analysis to assess the success of telemedicine visits and included the aforementioned demographic and clinical variables as potential confounders. Standard model diagnostics, including collinearity, variance inflation, tolerance, and goodness-of-fit tests, were examined. We performed a sensitivity analysis for the multivariate logistic regression model, analyzing only visits between May and July 2020 to limit the influence of patient and practitioner challenges with new technology and telemedicine platforms for visits at the beginning of the pandemic (March to April 2020). All analyses were performed using SAS, version 9.4 (SAS Institute), and RStudio, version 4.0.2 (RStudio, PBC), with 2-sided *P* values <.05 considered to indicate statistical significance.

## Results

### Demographic Data

We identified 759 patients with thoracic malignant tumors and 2106 total visits (Figure 1). A total of 39 patients and 75 visits were excluded because they did not have at least 1 completed office note with a medical oncologist. Of the remaining 2031 patient visits, 679 (33.43%) were in person and 1352 (66.57%) were telemedicine. Ninety-one telemedicine visits (6.73%) were then excluded because encounter data were not available, leaving 1261 analyzable telemedicine visits (65.00% of the 1940 total visits in the analysis), of which 717 (56.86%) were successful and 544 (43.14%) were unsuccessful (496 [91.18%] were conducted via telephone without video and 48 [8.82%] were no-show or missed encounters).

Demographic data for the 720 patients included in the analysis are shown in Table 1. The median age was 65.7 years (range, 54.7-76.7 years). Most patients were older than 65 years (402 [55.83%]), were male (384 [53.33%]), identified as White (511 [70.97%]), had private insurance (310 [43.06%]), and were married or partnered (461 [64.03%]). Missing data were minimal, with 91 of 1352 telemedicine visits (6.73%) missing success status, 2 (0.15%) missing cancer stage, and 2 (0.15%) missing type of therapy. Table 1 shows the association of demographic factors with odds of having had a telemedicine visit, given as GEE-adjusted ORs. Individuals who were married or partnered were more likely to be seen via telemedicine (OR, 1.26; 95% CI, 1.02-1.55). There was no statistically significant difference identified in any of the other demographic groups; however, there were significantly fewer telemedicine visits in March than from April to July. Patients receiving immunotherapy (OR, 0.60; 95% CI, 0.43-0.82) or no therapy (OR, 0.67; 95% CI, 0.50-0.89) had lower odds of being seen via telemedicine.

**Table 1.** Patient Demographics and Odds of In-Person vs Telemedicine Visits

Category	Unique patients, No. (%) (N = 720)	Visits, No./total No. (%) (N = 2031)^a^		GEE-adjusted OR for telemedicine (95% CI)
		In person (n = 679)	Telemedicine (n = 1352)	
Age, y				
>65	402 (55.83)	396/1146 (34.55)	750/1146 (65.45)	0.88 (0.72-1.08)
≤65	318 (44.17)	283/885 (31.98)	602/885 (68.02)	1 [Reference]
Sex				
Female	336 (46.67)	297/926 (32.07)	629/926 (67.93)	1 [Reference]
Male	384 (53.33)	382/1105 (34.57)	723/1105 (65.43)	0.88 (0.72-1.07)
Race				
Black	133 (18.47)	136/375 (36.27)	239/375 (63.73)	0.97 (0.67-1.40)
White	511 (70.97)	473/1448 (32.67)	975/1448 (67.33)	1 [Reference]
Other^b^	76 (10.56)	70/208 (33.65)	138/208 (66.35)	0.86 (0.67-1.12)
Ethnicity				
Hispanic or Latino	20 (2.78)	25/58 (43.10)	33/58 (56.90)	0.64 (0.30-1.35)
Not Hispanic or Latino	677 (94.03)	637/1930 (33.01)	1293/1930 (66.99)	1 [Reference]
Not specified or unknown	23 (3.19)	17/43 (39.53)	26/43 (60.47)	0.79 (0.46-1.36)
Insurance				
Medicare	274 (38.06)	271/771 (35.15)	500/771 (64.85)	0.87 (0.70-1.09)
Medicare plus supplementary insurance	89 (12.36)	75/237 (31.65)	162/237 (68.35)	1.00 (0.75-1.34)
Medicaid	47 (6.53)	49/125 (39.20)	76/125 (60.80)	0.72 (0.49-1.05)
Private or self-pay	310 (43.06)	284/898 (31.63)	614/898 (68.37)	1 [Reference]
Marital status				
Single, divorced, separated, widowed, or unknown	259 (35.97)	272/746 (36.46)	474/746 (63.54)	1 [Reference]
Married or partnered	461 (64.03)	407/1285 (31.67)	878/1285 (68.33)	1.26 (1.02-1.55)
Cancer type				
NSCLC	521 (72.36)	497/1512 (32.87)	1015/1512 (67.13)	1 [Reference]
Small cell lung	49 (6.81)	44/138 (31.88)	94/138 (68.12)	1.04 (0.72-1.51)
Esophageal	114 (15.83)	107/299 (35.79)	192/299 (64.21)	0.88 (0.66-1.17)
Other	36 (5.00)	31/82 (37.80)	51/82 (62.20)	0.82 (0.52-1.28)
Cancer stage				
I/II	107 (14.9**0**)	66/216 (30.56)	150/216 (69.44)	1 [Reference]
III	158 (22.01)	178/473 (37.63)	295/473 (62.37)	0.70 (0.48-1.03)
IV	453 (63.09)	434/1337 (32.46)	903/1337 (67.54)	0.91 (0.65-1.28)
Therapy type				
Chemotherapy	76 (10.58)	151/532 (28.38)	381/532 (71.62)	1 [Reference]
Combination	48 (6.69)	91/273 (33.33)	182/273 (66.67)	0.79 (0.53-1.17)
Immunotherapy	73 (10.17)	128/327 (39.14)	199/327 (60.86)	0.60 (0.43-0.82)
No therapy	411 (57.24)	245/657 (37.29)	412/657 (62.71)	0.67 (0.50-0.89)
Tyrosine kinase inhibitor	110 (15.32)	62/231 (26.84)	169/231 (73.16)	1.06 (0.73-1.56)
Month				
March	323 (44.86)	367/457 (80.31)	90/457 (19.69)	1 [Reference]
April	122 (16.94)	44/405 (10.86)	361/405 (89.14)	31.93 (22.67-44.97)
May	100 (13.89)	40/396 (10.10)	356/396 (89.90)	37.68 (25.40-55.89)
June	139 (19.31)	153/526 (29.09)	373/526 (70.91)	10.00 (7.55-13.26)
July	36 (5.00)	75/247 (30.36)	172/247 (69.64)	9.80 (6.83-14.06)
High-risk zip code^c^				
No	658 (91.39)	588/1799 (32.68)	1211/1799 (67.32)	1 [Reference]
Yes	62 (8.61)	91/232 (39.22)	141/232 (60.78)	0.74 (0.52-1.05)

Abbreviations: GEE, generalized estimating equation; NSCLC, non–small cell lung cancer; OR, odds ratio.

^a^ Percentages shown are for the proportion of in-person or telemedicine visits among the total visits of both types for the given demographic category.

^b^ Other includes American Indian or Alaska Native, Asian, Native Hawaiian or other Pacific Islander, or not specified.

^c^ Defined as zip codes in East Baltimore, Maryland, that had previously been identified through our institutional cancer registry as having elevated rates of cancer mortality compared with other zip codes in Baltimore.

### Odds of a Successful Telemedicine Visit

**Figure 2.**
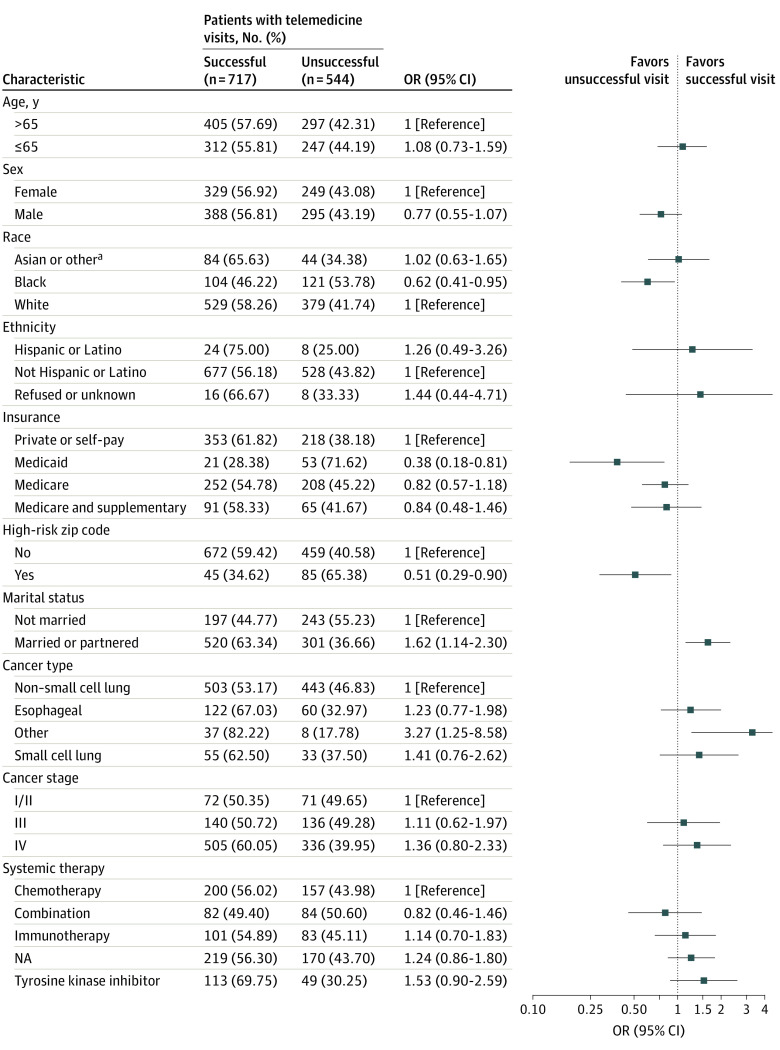
Odds Ratios (ORs) for Successful vs Unsuccessful Telemedicine Visits Percentages shown are for the proportion of successful or unsuccessful visits among the total telemedicine visits that occurred within the given demographic category. ^a^Other includes American Indian or Alaska Native, Native Hawaiian or other Pacific Islander, or not specified.

Figure 2 shows the results of the multivariate GEE-adjusted logistic regression analysis for the 1261 telemedicine visits included. Of note, patients who were Black (OR, 0.62; 95% CI, 0.41-0.95), had Medicaid (OR, 0.38; 95% CI, 0.18-0.81), or were from a high-risk zip code (OR, 0.51; 95% CI, 0.29-0.90) had lower adjusted odds of having successful telemedicine visits. Patients who were married or partnered (OR, 1.62; 95% CI, 1.14-2.30) were more likely to have successful telemedicine visits. A sensitivity analysis including only visits from May to July found similar results for high-risk zip codes and patients receiving tyrosine kinase inhibitor therapy, and there were associations in the same direction for race, ethnicity, insurance, and marital status.

### Association of Telemedicine Visit Success Status With Clinical Outcomes and Resource Use

Table 2 shows the rates of death, ED and urgent care visits, and hospitalization stratified by telemedicine visit types and by whether telemedicine visits were successful. Patients with a mix of successful telemedicine visits and more than 1 unsuccessful telemedicine visit (OR, 2.73; 95% CI, 1.42-5.22) and patients with no successful telemedicine visit despite having multiple telemedicine visits (OR, 3.43; 95% CI, 1.80-6.52) had higher odds of ED visits compared with patients with all successful telemedicine visits. Patients with only 1 unsuccessful telemedicine visit and the rest successful (OR, 2.81; 95% CI, 1.44-5.47), more than 1 unsuccessful telemedicine visit and the rest successful (OR, 4.50; 95% CI, 2.41-8.41), and more than 1 telemedicine visit with none successful (OR, 4.24; 95% CI, 2.24-8.03) had a higher likelihood of having an urgent care visit. Patients with only 1 telemedicine visit that was unsuccessful had a lower likelihood of having an urgent care visit (OR, 0.29; 95% CI, 0.10-0.85). In addition, the groups with 1 unsuccessful telemedicine visit and the rest successful (OR, 2.97; 95% CI, 1.50-5.88), more than 1 unsuccessful visit and the rest successful (OR, 2.37; 95% CI, 1.17-4.80), and more than 1 telemedicine visit and none successful (OR, 4.19; 95% CI, 2.17-8.10) had higher odds of hospitalization.

**Table 2.** Association of Telemedicine Visit Success With Clinical Outcomes

Success status^a^	Total visits, No.	Death		Any ED visit		Any urgent care visit		Any hospitalization	
		No. (%)	OR (95% CI)	No. (%)	OR (95% CI)	No. (%)	OR (95% CI)	No. (%)	OR (95% CI)
**Among telemedicine visits**									
All success	278	31 (11.15)	1 [Reference]	37 (13.31)	1 [Reference]	35 (12.59)	1 [Reference]	31 (11.15)	1 [Reference]
Mixed unsuccess 1	59	11 (18.64)	1.83 (0.86-3.88)	15 (25.42)	2.22 (1.12-4.39)	17 (28.81)	2.81 (1.44-5.47)	16 (27.12)	2.97 (1.50-5.88)
Mixed unsuccess >1	61	5 (8.20)	0.71 (0.27-1.91)	18 (29.51)	2.73 (1.42-5.22)	24 (39.34)	4.50 (2.41-8.41)	14 (22.95)	2.37 (1.17-4.80)
No success >1	58	12 (20.69)	2.08 (1.00-4.34)	20 (34.48)	3.43 (1.80-6.52)	22 (37.93)	4.24 (2.24-8.03)	20 (34.48)	4.19 (2.17-8.10)
No success 1	99	7 (7.07)	0.61 (0.26-1.43)	11 (11.11)	0.81 (0.40-1.67)	4 (4.04)	0.29 (0.10-0.85)	8 (8.08)	0.70 (0.31-1.58)
**Telemedicine visits vs in-person visits**									
In-person only	155	30 (19.35)	1 [Reference]	33 (21.29)	1 [Reference]	25 (16.13)	1 [Reference]	27 (17.42)	1 [Reference]
All success	278	31 (11.15)	0.52 (0.30-0.90)	37 (13.31)	0.57 (0.34-0.95)	35 (12.59)	0.75 (0.43-1.31)	31 (11.15)	0.60 (0.34-1.04)
Mixed unsuccess 1	59	11 (18.64)	0.96 (0.44-2.06)	15 (25.42)	1.26 (0.63-2.54)	17 (28.81)	2.11 (1.04-4.27)	16 (27.12)	1.76 (0.87-3.58)
Mixed unsuccess >1	61	5 (8.20)	0.37 (0.14-1.01)	18 (29.51)	1.55 (0.79-3.03)	24 (39.34)	3.37 (1.73-6.58)	14 (22.95)	1.41 (0.68-2.92)
No success >1	58	12 (20.69)	1.09 (0.51-2.30)	20 (34.48)	1.95 (1.00-3.78)	22 (37.93)	3.18 (1.61-6.28)	20 (34.48)	2.50 (1.26-4.94)
No success 1	99	7 (7.07)	0.32 (0.13-0.75)	11 (11.11)	0.46 (0.22-0.96)	4 (4.04)	0.22 (0.07-0.65)	8 (8.08)	0.42 (0.18-0.96)

Abbreviations: ED, emergency department; OR, odds ratio.

^a^ All success included patients for whom all telemedicine visits were successful; mixed unsuccess 1, patients who had 1 unsuccessful telemedicine visit but all the others were successful; mixed unsuccess >1, patients who had more than 1 unsuccessful telemedicine visit but the others were successful; no success >1, patients who had more than 1 telemedicine visit and all were unsuccessful; no success 1, patients who had 1 unsuccessful telemedicine visit and no other telemedicine visits during the study period; and in-person only, patients who only had in-person visits during the study period.

Compared with the in-person visit–only group, patients with all successful telemedicine visits had lower odds of ED visits (OR, 0.57; 95% CI, 0.34-0.95) and death (OR, 0.52; 95% CI, 0.30-0.90). Similarly, the group with no successful telemedicine visits but with only 1 telemedicine visit overall had lower odds of ED visits (OR, 0.46; 95% CI 0.22-0.96), urgent care visits (OR, 0.22; 95% CI, 0.07-0.65), hospitalizations (OR, 0.42; 95% CI 0.18-0.96), and death (OR, 0.32; 95% CI, 0.13-0.75) compared with the in-person visit–only group. The group with only 1 unsuccessful telemedicine visit and the rest successful (OR, 2.11; 95% CI, 1.04-4.27), the group with more than 1 unsuccessful telemedicine visit and the rest successful (OR, 3.37; 95% CI, 1.73-6.58), and the group with no successful telemedicine visits among more than 1 telemedicine visit (OR, 3.18; 95% CI, 1.61-6.28) had higher odds of urgent care visits compared with the in-person visit–only group. The group with no successful telemedicine visits among more than 1 telemedicine visit had higher odds of hospitalizations (OR, 2.50; 95% CI, 1.26-4.94) compared with the in-person visit–only group.

### Association of Telemedicine Visit Success With Changes in Systemic Cancer Treatment

Table 3 shows the ORs for having a telemedicine visit and for having a successful telemedicine visit among various changes of therapy. Patients who were starting therapy had lower GEE-adjusted odds of having a telemedicine visit compared with an in-person visit (OR, 0.49; 95% CI, 0.37-0.64) and higher GEE-adjusted odds of having a successful telemedicine visit compared with an unsuccessful visit (OR, 1.90; 95% CI, 1.28-2.82). In addition, patients with a switch in therapy had higher GEE-adjusted odds of having a successful telemedicine visit (OR, 1.86; 95% CI, 1.08-3.22). We did not observe any statistically significant differences in the odds of having a telemedicine visit or in the odds of a successful telemedicine visit among patients who experienced a delay in therapy or dose modification.

**Table 3.** Association of Telemedicine Visit Success With Changes in Systemic Cancer Treatment

Change in therapy	Visits, No. (%)		OR for telemedicine (95% CI)	Visits, No. (%)		OR for successful telemedicine visit (95% CI)
	In person (n = 658)	Telemedicine (n = 1294)		Successful telemedicine (n = 717)	Unsuccessful telemedicine (n = 527)	
Delay in therapy	44 (6.69)	95 (7.34)	0.97 (0.66-1.42)	51 (7.11)	39 (7.40)	1.06 (0.68-1.65)
Dose increase	1 (0.15)	1 (0.08)	0.45 (0.03-7.19)	1 (0.14)	0	NA
Dose reduction	12 (1.82)	29 (2.24)	1.08 (0.54-2.15)	12 (1.67)	14 (2.66)	0.69 (0.32-1.52)
Hospice	14 (2.13)	20 (1.55)	0.64 (0.32-1.28)	15 (2.09)	4 (0.76)	3.04 (1.00-9.24)
Maintenance	311 (47.26)	694 (53.63)	1 [Reference]	367 (51.19)	297 (56.36)	1 [Reference]
Start of therapy	129 (19.60)	141 (10.9)	0.49 (0.37-0.64)	96 (13.39)	41 (7.78)	1.90 (1.28-2.82)
Surveillance	117 (17.78)	247 (19.09)	0.95 (0.73-1.22)	129 (17.99)	112 (21.25)	0.93 (0.69-1.25)
Switch in therapy	30 (4.56)	67 (5.18)	1.00 (0.64-1.57)	46 (6.42)	20 (3.80)	1.86 (1.08-3.22)

Abbreviation: OR, odds ratio.

## Discussion

One of the many impacts of the COVID-19 pandemic was the need to quickly change to a telemedicine model, with a rapid increase from 20% of visits at our institution in March 2020 being scheduled as telemedicine to 90% in April and May. Individuals in this study who were married or with a partner were more likely to have a telemedicine visit, but there were no statistically significant differences in the odds of telemedicine visits vs in-person visits in other demographic groups. Patients who were Black; had Medicaid; were single, divorced, separated, or widowed; and were from high-risk zip codes were less likely to successfully complete telemedicine visits than to have unsuccessful visits. We found an association between telemedicine success and ED and urgent care visits and hospitalization. Furthermore, we found that starting a new therapy was associated with lower odds of having a telemedicine visit than an in-person visit but a higher likelihood of success of telemedicine; a switch in therapy was also associated with higher likelihood of success of telemedicine.

Our results are generally concordant with those of other studies identifying sociodemographic factors associated with disparities in the success of telemedicine. Those studies identified Black race^9,10,11,12,13^ and Medicaid insurance as factors associated with disparities in telemedicine success.^11,16,17^ However, we identified additional factors associated with telemedicine success, such as being married or having a partner. Being married or having a partner may increase the chance that the patient has someone else to assist with completing telemedicine visits at home. Patients receiving immunotherapy were more likely to be seen in person possibly because of clinicians wanting to evaluate patients for immune-related adverse effects.

In a recent study of patients with head and neck cancer, investigators reported that patients with Medicaid or other public insurance (OR, 0.26; 95% CI, 0.10-0.66) and low median household income had lower completion of virtual oncology visits.^18^ These authors stated that although “synchronous audio and visual communication in virtual visits offer a more comprehensive assessment, telephone visits may be an important avenue to access care.” However, we categorized telephone-only visits as an unsuccessful telemedicine visit. We hypothesized that important clinical information could be missed via audio-only connections, and video visits were considered both the institutional and the Medicare standard to replace in-person visits, with limited reimbursement given for audio-only visits.^19^ Subsequent studies have used a similar method to identify unsuccessful visits,^6,20^ and video visits, rather than telephone only, were rated as highly as in-person visits.^21^ Inability to complete audio and visual assessments may be associated with some of the negative outcomes we observed or may be a surrogate for a more disadvantaged patient population at risk of inferior outcomes.

To our knowledge, the present study is one of the first to analyze successful use of telemedicine visits and its association with clinical outcomes and resource use in the form of ED and urgent care visits and hospitalizations. Compared with having all successful telemedicine visits, having an unsuccessful visit was associated with a higher risk of hospitalization or an ED or urgent care visit except when only 1 telemedicine visit occurred during the study period and was unsuccessful. Most of the patients with only 1, unsuccessful visit were those undergoing surveillance imaging, and therefore, they only needed to be seen once during the study period. For example, patients with stable disease undergoing periodic computed tomography scans (eg, those receiving highly effective tyrosine kinase inhibitors) may be less likely to have complications and could more easily have their visits conducted by telephone. Similarly, patients with all successful telemedicine visits had lower odds of death compared with patients who had in-person visits only. Some visits may have been changed to telephone only because of the clinical setting rather than owing to patient access factors, or healthier patients may have been preferentially seen via telemedicine. Schmidt et al^10^ assessed cancer care outcomes, including COVID-19 and treatment delays owing to COVID-19, and reported that more non-Hispanic Black and Hispanic patients diagnosed with COVID-19 and more Hispanic patients had treatment delays compared with White patients. They did not report outcomes for resource use, such as ED or urgent care visits or hospitalizations. Our analysis did not identify any treatment delays associated with telemedicine visits, and the association we found between starting new therapy and lower use of telemedicine was likely because of the clinical practice of seeing patients at the time of treatment initiation. The findings of resource use differences are, to our knowledge, unique to our analysis and are important clinical outcomes in care delivery.

### Limitations

This study has limitations. Our analysis was retrospective; thus, collection of demographic and clinical factors was dependent on accurate medical record documentation. In addition, at the beginning of the pandemic, both patients and practitioners were accommodating to new technology and likely had challenges accessing telemedicine platforms. This likely contributed to more unsuccessful visits in March and April 2020 than in later months, and whether visits were successful or not may not have been accurately documented or captured early in the pandemic. However, because our sensitivity analyses limited to visits in later months showed similar results, these technological challenges may not have had as large an impact as anticipated. Furthermore, we categorized both no-show visits and telephone-only visits as unsuccessful owing in part to sample size limitations, although clinically, having a telephone-only visit would likely still be better than a no-show visit. Also, most of the data during visits relies on collection of encounters in the electronic medical record and clinicians’ notes, which may be inaccurate or not up to date (eg, we could not capture outcome data from outside hospitals or clinics). We were only able to capture outcomes available in Epic through Johns Hopkins–affiliated hospitals or integrated electronic medical records; thus, we anticipate there were additional events that we were not able to capture, potentially producing nondifferential misclassification bias. We also believe that the potential misclassification of outcomes was nondifferential and may have biased our findings toward the null hypothesis. In addition, an association with inferior outcomes is hypothesis generating but does not prove causality between telemedicine success and clinical outcomes, leading to an increased risk of type I error. Furthermore, we could not establish an exact temporal association preceding each clinical outcome.

## Conclusions

In this cohort study, patients with thoracic cancer who were Black, had Medicaid, or were from a high-risk zip code had increased odds of unsuccessful telemedicine visits and unsuccessful telemedicine visits were associated with worse clinical outcomes. These findings suggest that disparities exist in access to and successful completion of telemedicine visits for patients with cancer. We have begun to characterize the outcomes associated with this lack of access, and more studies are needed to understand the association between telemedicine success and outcomes. Telemedicine should be used to provide more health care access to individuals who are otherwise disadvantaged, and new digital health innovations should serve to decrease the digital divide rather than add to it. Future studies that longitudinally assess the association between sociodemographic factors, telemedicine access, and clinical outcomes may serve as a foundation for more personalized care and development of targeted, risk-adapted interventions for those at risk of inferior health outcomes. These types of studies will be crucial to strive toward “techquity” in oncology care.

## Notes

Supplement. **eFigure.** High-risk Zip Codes in East Baltimore
