# Supplementary material for: Disparities in Telemedicine Success and Their Association With Adverse Outcomes in Patients With Thoracic Cancer During the COVID-19 Pandemic
Source: JAMA Netw Open. 2022 Jul 7;5(7):e2220543. doi: 10.1001/jamanetworkopen.2022.20543 (PMC9264040; doi:10.1001/jamanetworkopen.2022.20543)
Supplement: Supplement. — eFigure. High-risk Zip Codes in East Baltimore [file jamanetwopen-e2220543-s001.pdf]

Supplementary Online Content

Waseem N, Boulanger M, Yanek LR, Feliciano JL. Disparities in telemedicine success and their association with adverse outcomes in patients with thoracic cancer during the COVID-19 pandemic. *JAMA Netw Open*. 2022;5(7):e2220543. doi:10.1001/jamanetworkopen.2022.20543

eFigure. High-risk Zip Codes in East Baltimore

This supplementary material has been provided by the authors to give readers additional information about their work.

© 2022 Waseem N et al. *JAMA Network Open*.

eFigure. High-risk Zip Codes in East Baltimore

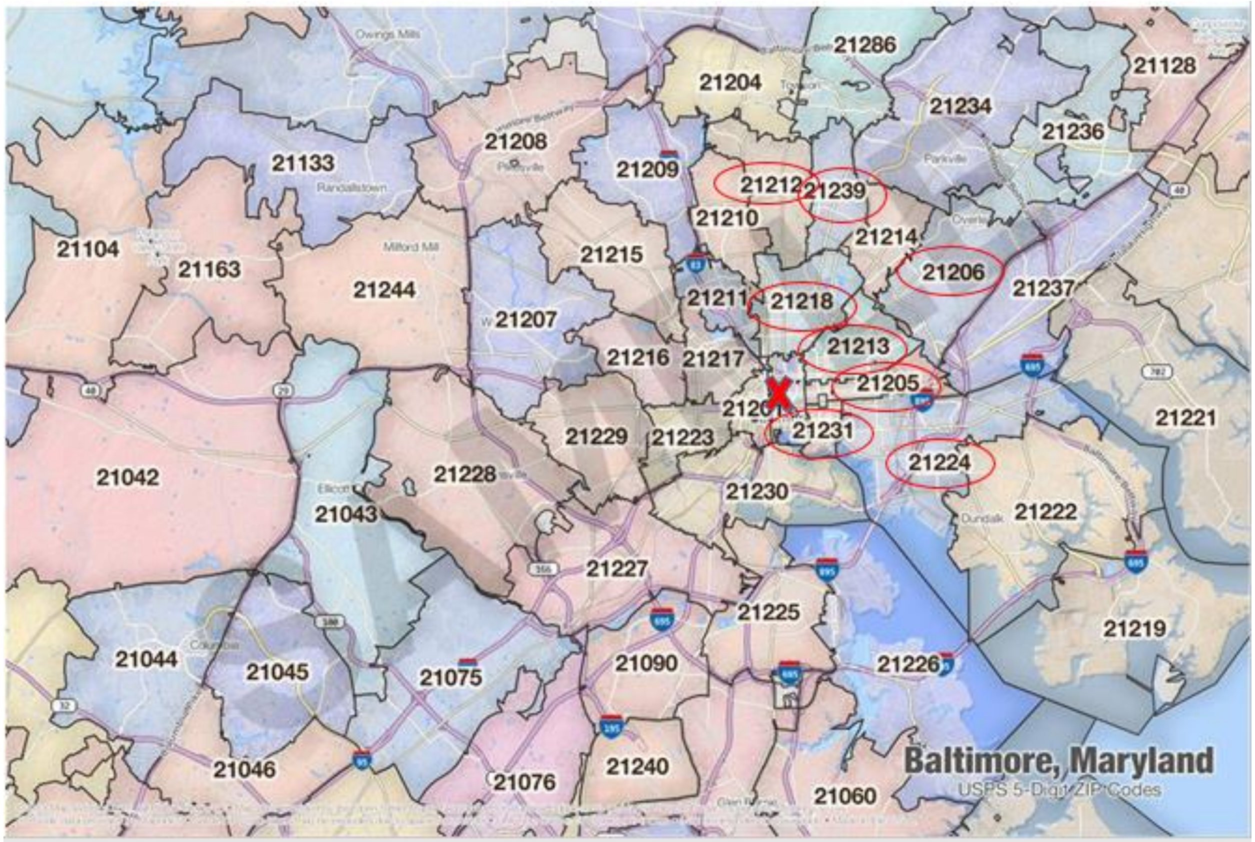

Zip codes including 21205, 21206, 21212, 21213, 21218, 21224, 21231, 21239. X is the approximate location of Johns Hopkins Hospital

© 2022 Waseem N et al. *JAMA Network Open*.
